# Increased RNAi Efficacy in *Spodoptera exigua* via the Formulation of dsRNA With Guanylated Polymers

**DOI:** 10.3389/fphys.2018.00316

**Published:** 2018-04-04

**Authors:** Olivier Christiaens, Myriam G. Tardajos, Zarel L. Martinez Reyna, Mamoni Dash, Peter Dubruel, Guy Smagghe

**Affiliations:** ^1^Laboratory of Agrozoology, Department of Crop Protection, Ghent University, Ghent, Belgium; ^2^Polymer Biochemistry and Biomaterials Group, Department of Organic and Macromolecular Chemistry, Faculty of Sciences, Ghent University, Gent, Belgium

**Keywords:** RNA interference, dsRNA degradation, dsRNA-polymer nanocarriers, crop protection, Lepidoptera, *Spodoptera exigua*

## Abstract

Lepidoptera comprise some of the most devastating herbivorous pest insects worldwide. One of the most promising novel pest control strategies is exploiting the RNA interference (RNAi) mechanism to target essential genes for knockdown and incite toxic effects in the target species without harming other organisms in the ecosystem. However, many insects are refractory to oral RNAi, often due to rapid degradation of ingested dsRNA in their digestive system. This is the case for many lepidopteran insects, including the beet armyworm *Spodoptera exigua*, which is characterized by a very alkaline gut environment (pH > 9.0) and a strong intestinal nucleolytic activity. In this research, guanidine-containing polymers were developed to protect dsRNA against nucleolytic degradation, specifically in high pH environments. First, their ability to protect dsRNA against nucleolytic degradation in gut juice of the beet armyworm *S. exigua* was investigated *ex vivo*. Polymers with high guanidine content provided a strong protection against nucleolytic degradation at pH 11, protecting the dsRNA for up to 30 h. Next, cellular uptake of the dsRNA and the polyplexes in lepidopteran CF203 midgut cells was investigated by confocal microscopy, showing that the polymer also enhanced cellular uptake of the dsRNA. Finally, *in vivo* feeding RNAi bioassays demonstrated that using these guanidine-containing polymer nanoparticles led to an increased RNAi efficiency in *S. exigua*. Targeting the essential gene *chitin synthase B*, we observed that the mortality increased to 53% in the polymer-protected dsRNA treatment compared to only 16% with the naked dsRNA and found that polymer-protected dsRNA completely halted the development of the caterpillars. These results show that using guanylated polymers as a formulation strategy can prevent degradation of dsRNA in the alkaline and strongly nucleolytic gut of lepidopteran insects. Furthermore, the polymer also enhances cellular uptake in lepidopteran midgut cells. This new delivery strategy could be of great use in further fundamental research in lepidopterans, using RNAi as a research tool, and could lead to future applications for RNAi-based pest control of lepidopteran insects.

## Introduction

Over the past few decades, crop protectors have been looking for novel and intelligent ways to combat insect pests as an alternative to broad spectrum and chemical pesticides that are still being used copiously these days. One of the most promising strategies that could have a significant economic impact is the use of RNA interference (RNAi). This technology, based on post-transcriptional gene silencing, allows for a very species-specific targeting of insect pests without harming non-target organisms, including beneficial insects such as predators and pollinators. Furthermore, the environmental fate of the active biomolecules, double-stranded RNA (dsRNA), is thought to be short-lived since dsRNAs are shown to be degraded in soil in a matter of days (Dubelman et al., 2014). The biological mechanism is based on the introduction of this dsRNA, specific to an essential target gene, into the cells of the target species upon oral ingestion, where it is then taken up into the RNAi machinery and eventually leads to the degradation of the target gene's messenger RNA (mRNA), effectively knocking down its expression. Over the past few years, the first commercial products have been developed and are now close to commercialization in North-America. One of these is a transgenic corn event expressing a dsRNA targeting the *Snf7* gene of the Western corn rootworm, *Diabrotica virgifera* (Baum et al., 2007; Bolognesi et al., 2012; Bachman et al., 2013). While this particular strategy, aimed to target larvae living within the roots, has been developed to be implemented in transgenic crops, an alternative way of application, targeting insects feeding on the green parts of the crops could be spraying dsRNA on the crops. Several research papers have demonstrated the potential success of this delivery method (Zhu et al., 2011; Gong et al., 2013; Kwon et al., 2013) and the first products, for example targeting the Colorado potato beetle *Leptinotarsa decemlineata*, are also under commercial development.

Despite its great efficacy in controlling certain insect pests, e.g., many beetle species, RNAi efficiency in insect species can be very variable. Many caterpillars (Lepidoptera) for example seem to be very refractory to RNAi, especially upon oral delivery of dsRNA (Arimatsu et al., 2007; Terenius et al., 2011; Liu et al., 2012; Garbutt et al., 2013). Several factors have been proposed to explain this variability between different insect species and insect orders. The two most important causes are most likely an impaired or slow cellular uptake of dsRNA in the gut and degradation of the dsRNA in the digestive tract of insects (Christiaens and Smagghe, 2014). In fruit flies for example, it has been shown that uptake efficiency is low, possibly due to the lack of Sid1-like channel proteins, which constitutes one of the two major dsRNA-uptake mechanisms that are found in most insects (Saleh et al., 2006; Ulvila et al., 2006; Miller et al., 2008; Tomoyasu et al., 2008; Whyard et al., 2009; Cappelle et al., 2016; Taning et al., 2016). In fact, in *Drosophila melanogaster* and *Drosophila suzukii* fruit flies, encapsulating the dsRNA in liposomic particles resulted in a much higher RNAi efficiency (Whyard et al., 2009; Taning et al., 2016). The other major factor involved in variable RNAi efficiency, degradation of dsRNA in the insect body, has been proven in several insect species as well, including lepidopteran, hemipteran, and orthopteran species (Allen and Walker, 2012; Garbutt et al., 2013; Luo et al., 2013; Christiaens et al., 2014; Wynant et al., 2014). Furthermore, in the lepidopteran silkmoth *Bombyx mori*, a DNA/RNA non-specific nuclease, capable of degrading dsRNA, was identified in the gut, associated with a very low efficiency of oral RNAi (Arimatsu et al., 2007; Liu et al., 2012). Lepidoptera in general, also exhibiting a very alkaline pH in the gut environment, are notorious for their strong and fast dsRNA-degrading capacity (Terenius et al., 2011; Garbutt et al., 2013). In order to develop an efficient RNAi-based method to control these economically very important pest organisms, formulations are required that can protect the dsRNA against this nucleolytic degradation and prolong the stability of the dsRNA in the gut long enough to allow sufficient cellular uptake by the midgut cells.

In human medicine and drug formulation, polymers for gene delivery application have been used extensively to protect nucleic acids in harsh environments (Ji et al., 2011; Lin et al., 2013; Cavallaro et al., 2017; Jones et al., 2017). In insects, a proof of concept has been reported when the natural polymer chitosan was used to complex dsRNA, thereby improving the RNAi-efficiency in mosquitoes (Zhang et al., 2010). In another study, a fluorescent cationic core-shell nanoparticle, consisting of a fluorescent core and polymer shells terminating with multiple amino groups was designed for *Drosophila* RNAi delivery (He et al., 2013). However, most polymers that have been used in nanoparticles so far, in mammals or in insects, were not expected to protect dsRNA in strong alkaline environments as those found in the gut of most Lepidoptera. Therefore, polymers specifically designed to be able to withstand decomplexation at high pH are necessary. In view of this, polymers containing strong basic side groups such as guanidine (pK_a_ 13.6) in their structure are interesting candidates. Other guanidine-containing polymers have previously been investigated in DNA delivery studies with mammalian systems and have proved to be not only able to complex the nucleotides but also to improve the transfection efficiency due to the guanidine functionalities present (Funhoff et al., 2004; Choi et al., 2015; Guo et al., 2015). Nevertheless, their potential as nucleotide protectors in an alkaline medium has never been analyzed.

In this study, a simple route to synthesize several cationic polymethacrylate derivatives carrying guanidine functionality was designed with the aim to form stable complexes with dsRNA under high pH environments while preventing its nucleolytic degradation. Investigation of the complexation of dsRNA and polymer was performed to determine the necessary charge ratio for full complexation. Next, a polymer preselection was made by conducting *ex vivo* degradation assays, using gut juice from *Spodoptera exigua*, at pH 7.5 and 11. This allowed us to screen for polymers that were able to protect dsRNA in alkaline environments. The cellular uptake of the dsRNA and the polymer-dsRNA complexes were then investigated by use of confocal microscopy. Finally, the best protecting polymer at pH 11 was selected for *in vivo* feeding experiments with *S. exigua* larvae, where the essential gene *chitin synthase B* was used as a target gene to evaluate RNAi efficiency.

## Materials and methods

### Materials

2-(dimethylamino) ethylmethacrylate (DMAEMA, Sigma- Aldrich) was distilled under reduced pressure prior to use. 1H-pyrazole-1-carboxamidine hydrochloride, (HPC, Sigma-Aldrich), N-(2aminoethyl) methacrylate hydrochloride (AEMA, Polysciences), sodium dihydrogen phosphate (NaH_2_PO_4_, Sigma-Aldrich), triethylamine (TEA, Sigma-Aldrich), acetonitrile (CH_3_CN, Sigma-Aldrich), ammonium persulfate (APS), Fluorescein isothiocyanate (FICT), were used as received.

### Polymer synthesis, modification, and characterization

#### Polymer synthesis

Monomers (AEMA and/or DMAEMA) and initiator (ammonium persulfate, APS) were dissolved in water (pH 4) in a total concentration of 0.5–1 mol L^−1^ and 8.7 × 10^−1^ mol L^−1^, respectively. Nitrogen was flushed through the polymerization solution for 30 min. Then polymerization was carried out at 70°C during 24 h. The formed polymer or copolymer was isolated and purified by dialysis against MilliQ water in 3,500 g/mol cut-off membranes (48 h) followed by freeze-drying. The nomenclature for the different polymers used in this study is given in Table 1.

**Table 1:** Overview of synthesized polymers and their guanylated derivatives.

**(co)polymer**	**Code**	**Guanylated polymer**	**Code**
PAEMA High Mw	PAH	P(AEMA_0.13_-co-GUMA_0.87_) High Mw	PAG 87H
		P(AEMA_0.67_-co-GUMA_0.33_)	PAG33
PAEMA Low Mw	PAL	P(AEMA_0.13_-co-GUMA_0.87_) Low Mw	PAG 87L
PDMAEMA	PD		
P(DMAEMA_0.52_-co-AEMA_0.48_)	PDA48	P(DMAEMA_0.52_-co-AEMA_0.30_-co-GUMA_0.18_)	PDAG18
P(DMAEMA_0.30_-co-AEMA_0.70_)	PDA70	P(DMAEMA_0.30_-co-AEMA_0.32_-co-GUMA_0.38_)	PDAG38

#### Polymer guanylation

Part of the primary amines from the AEMA moieties of the synthesized polymers were guanylated post-polymerization. The reaction was conducted in an aqueous solution for 24 h at room temperature with equivalent molar amounts of HPC and TEA to target the primary amines of AEMA. Then, the modified polymers were purified by dialysis (cut-off 3,500 g/mol) against deionized water for 48 h before freeze-drying. For fluorescent labeling of the primary amino groups, 30 mg of PAG87L was dissolved in a 0.1 M carbonate buffer (pH 8.3) and reacted with fluorescein isothiocyanate (FITC). FITC dissolved in dimethylformamide, was added dropwise at a molar ratio of 0.1 mol dye per mol of amino side groups, and incubated under continuous stirring for 3 h in the dark at room temperature. Unreacted FITC and salts were removed by dialysis against water for 48 h after which the polymer was isolated by freeze-drying.

#### Polymer characterization

Structural characterization of the polymers was carried out by ^1^H-NMR spectroscopy. ^1^H-NMR analysis of the (co)polymers was performed using a Bruker Avance-400 spectrometer in D_2_O with tetramethylsilane (TMS) as the internal standard. The analysis of the composition of the synthesized polymers was carried out by comparison of the integrated intensities of resonance signals with chemical shift at δ = 3.33 ppm (C*H*_2_-NH_2_) (AEMA) and δ = 3.50 ppm [C*H*_2_-N(CH_3_)_2_] (DMAEMA). After modification by guanylation, the analysis was carried out by comparison of the integrated intensities of resonance signals with chemical shifts at δ = 2.96 ppm (C*H*_2_-NH_2_) (AEMA), δ = 3.13 ppm [C*H*_2_-N(CH_3_)_2_] (DMAEMA), and δ = 3.51 ppm (C*H*_2_-NH-C-) (GUMA), respectively. Fourier-transform infrared spectroscopy (FTIR) of the polymers was performed on a Bio-Rad FTIR spectrometer FTS 575C with a resolution of 45 cm^−1^ operating in attenuated total reflection mode. The recorded spectra were analyzed using WIN-IR Pro software. The molecular weight of the synthesized polymers was analyzed by size-exclusion chromatography, SEC (Water 600 controller) with an isocratic pump (Millipore-Waters 510 pump) connected to a differential refractometric detector (Waters 410). Two aqueous columns (Shodex OHpak SB- 806m HQ) were conditioned at 25°C and used to elute the samples with mobile phase 4% NaH_2_PO_4_ and 3% CH_3_CN buffered solution at pH 4 at 1 mL min^−1^. Calibration of SEC was carried out with monodisperse dextran standards.

### dsRNA synthesis

First, RNA was extracted from *S. exigua* larvae using the Qiagen RNeasy Mini Kit, following the manufacturer's instructions. Quality of RNA was evaluated by 1.5% agarose gel electrophoresis and the quantity was measured using the DeNovix DS11 spectrophotometer. Next, 1 μg of RNA was used to synthesize cDNA, using Superscript II reverse transcriptase (Thermo Scientific). Primers were designed for the amplification of the *chitin synthase B* dsRNA-template, containing T7 promotor sequences at the 5′ end, by PCR. The primers are given in the Supplementary Table 1. Taq polymerase (Thermo Scientific) was used for this PCR. Each 50 μL PCR reaction contained 5 μL of Buffer, 1.5 μL of MgCL_2_, 1 μL of dNTP (10 μM), 1.25 μL of each primer (10 μM), 0.25 μL Taq polymerase and 34.75 μL of nuclease-free water. PCR conditions were 2' 94°C, 5x (30″ 94°C, 30″ 60°C, 30″ 72°C), 30x (30″ 94°C, 30″ 65°C, 30″ 72°C) and 3′ 72°C. After PCR, the template was purified using the E.Z.N.A. Cycle Pure kit (Omega Biotek). Finally, dsRNA synthesis was performed using the RNAi Megascript kit (Thermo Scientific), following the manufacturer's instructions and using nuclease-free water for elution, rather than the provided elution solution. DsRNA specific for the gene encoding green fluorescent protein (GFP) in jellyfish was used as control. Primers for dsGFP synthesis are given in the Supplementary Table 1.

### Polymer-dsRNA complexation

The stoichiometry of the polymer-dsRNA complex formation in water was defined as the polymer-dsRNA charge ratio (N/P ratio), which was calculated as the molar ratio of the polymer amino charges (N) to the phosphate (P) groups of dsRNA. A dsRNA mass per charge of 325 Da was used. The mass per charge of the copolymers was calculated assuming protonation of the amines of the AEMA, DMAEMA, and GUMA side groups. Polymer/dsRNA complexes were formed by adding polymer solutions of predetermined concentrations to an equal volume of a dsRNA solution (20 μg/mL dsRNA) to obtain a range of N/P ratios between 0.2:1 and 5:1. The solution was then mixed by vortexing for 10 s followed by incubation at room temperature for 20 min. The size and zeta potential of the formed complexes were measured by dynamic light scattering (DLS) using a Malvern Zetasizer Nano-ZS apparatus (Malvern Instruments Ltd.) operating at 4 mW He–Ne laser at 633 nm using disposable cuvettes. Agarose gel electrophoresis was also used to determine the N/P ratio necessary to have full complexation of the dsRNA. The formed polyplexes were run on a 1.5 % agarose gel. Gels were stained in an aqueous ethidium bromide solution (0.5 mg/L) for 30 min at room temperature.

### Insect rearing

Insects were taken from a continuous insect culture at the Lab of Agrozoology (Ghent University), which is maintained at 25°C and a 16:8 h light:dark photoperiod. The insects are kept on an artificial diet, as described earlier (Smagghe and Degheele, 1994).

### Degradation assays *ex vivo*

Entire midguts from L4 individuals were dissected and collected in 100 μL of 1x PBS at different pH-values (pH 7.5 and pH 11). These were stored on ice before subsequently being centrifuged for 10 min at 20.000 × g. Next, the supernatant was centrifuged again for 10′ at 20.000 × g. To investigate *ex vivo* degradation, 50 μL of polymer:dsRNA complex at a 4:1 N/P ratio and 30 μg/μL dsRNA concentration was added to 35 μL of midgut juice and samples were taken after incubation for different time points (1–30 h). As a control, naked dsRNA was incubated in the gut juice for 1 h. The polymer:dsRNA complex was decomplexed by adding 30 μL of a 1% SDS solution followed by 15 min at room temperature before loading on the gel. This SDS is added, not only to decomplex the polymer:dsRNA complex, but it also stops the enzymatic reaction of the nucleases in the midgut juice. Samples were then stored at −80°C until all samples were collected and loaded on a 1.5% agarose gel. For PAG87L, the same protocols were used, but multiple ratios and longer time points were analyzed.

### RNAi feeding bioassays

The PAG87L polymer was selected as nanocarrier in the *in vivo* feeding bioassays. Chitine synthase B (ChSB) was chosen as a target gene for RNAi in *S. exigua*. Polymer:dsRNA complexes were formed at a 2:1 N/P ratio. One hundred microliters of a 500 ng dsRNA/μL solution was coated on a 2 × 2 cm Chinese cabbage leaf disc, for both naked and polyplex treatments. Water, dsGFP and polymer (same concentration as in the polyplex treatment) were used as control treatments. Ten larvae were placed on each treated leaf disc. Leaf discs were replaced daily with newly treated fresh leaves for another 7 days. After day 7, untreated leaves were used to feed the insects. Mortality and the developmental stage of the larvae were recorded daily for 13 days and weight of the larvae was recorded every 2 days over the course of the experiment. The experiment was done three times in total (*N* = 30 per treatment for each repeat). Data on the developmental rate was collected over two repetitions of the experiment (*N* = 30 per treatment for each repeat). A Kruskal–Wallis with *post-hoc* Games-Howell statistical test for the mortality data and a one-way ANOVA with *post-hoc* Tuckey HSD statistical analysis for the weight data was performed using the SPSS software.

### Quantitative RT-PCR

Samples for transcriptional analysis were collected after 5 days of feeding on the treated Chinese cabbage leaves. Total RNA extraction was performed using the Qiagen RNeasy Mini Kit, following the manufacturer's instructions. Quality of RNA was evaluated by 1.5% agarose gel electrophoresis and the quantity was measured using the DeNovix DS11 spectrophotometer. Next, 1 μg of RNA was used to synthesize cDNA, using Superscript II reverse transcriptase (Thermo Scientific). The qPCR was performed on a CFX™ Real-Time PCR Detection System (BioRad) using GoTaq® qPCR master mix (Promega, Madison, WI). Amplification conditions were as follows: 5′ at 95°C, 39x (30″ at 95°C + 1′ at 60°C) finished by a melt curve analysis (65 to 95°C at 5″ 0.5°C increment). Actin, GAPDH and PGCP were used as reference genes and Cq-values were transformed into normalized relative expression taking the expression of the three reference genes into account (Hellemans et al., 2007). Primers were designed using Primer 3 and are given in the Supplementary Table 1, together with their amplification efficiency and *R*^2^-value. Statistical analysis for the qPCR data was performed on three biological samples per treatment (5–8 individual insects were pooled together per biological sample), using a One-way ANOVA and *post-hoc* Tuckey HSD test in SPSS.

### Confocal microscopy

The CF203 cell line, originating from the larval midgut of the lepidopteran insect *Choristoneura fumiferana* was selected for these confocal microscopy experiments. CF203 cells were cultured at 27°C in Insect-Xpress w/L-Gln containing 2.5% FBS and sub-cultured every 7 days in a 1:10 dilution. The day of the experiment, a Trypan blue exclusion test of cell viability was performed using a Bürker counting chamber. Five microliters of 0.4% trypan blue solution was added to 5 μL of cell suspension and observed under the inverted microscope.

DsRNA was labeled using the Label IT® siRNA Tracker Intracellular Localization Kit, Cy®3 (Mirus Bio) according to manufacturer's instructions. Each reaction allowed for the labeling of 10 μg of dsRNA. The concentration of the final stock solution was 500 ng/μL. Samples were kept in the dark during the complete protocol and stored at −20°C until used. The PAG87L polymer was labeled with FITC.

The cell suspension (~10^6^ cells/ml) was incubated with two different treatments: In the first treatment, 5.23 μL FITC-polymer was first mixed with 15 μL Cy3-dsRNA and 9.8 μL nuclease free-water. After 30 min, 30 μL Cy3-FITC-polyplex was added to 120 μL cell suspension. The final concentration of de dsRNA and polymer was 50 ng/μL. The samples were then incubated at 27°C for 10 min. After incubation, samples were centrifuged at 700 RPM for 1 min and the supernatant was removed without disturbing the pellet. To dye the nucleus, Hoechst® 33342 (Thermo Fisher Scientific) was used. The staining solution was prepared by diluting the Hoechst stock solution 1:2,000 in PBS. Fifty microliters diluted Hoechst solution was added to each vial and then incubated for 30 min at 27°C. Following this step, the samples were centrifuged at 700 RPM for 1 min and washed with PBS 3 times before resuspending the pellet in the appropriate growth medium. As a control treatment, naked dsRNA, labeled with Cy3, was added to the cell suspension using the same protocol.

For the confocal microscope analysis, 10 μL of each sample was pipetted on Ground Edges Microscope slides (VWR) and covered with a coverslip. The edges were sealed with nail polish. Three different excitation lasers were needed for this experiment: 352 nm for Hoechst® 33342, 495 nm for FITC and 549 nm for Cy3. Each picture is the results of an average of 4 shots.

## Results

### Polymer synthesis

**Figure 1:**
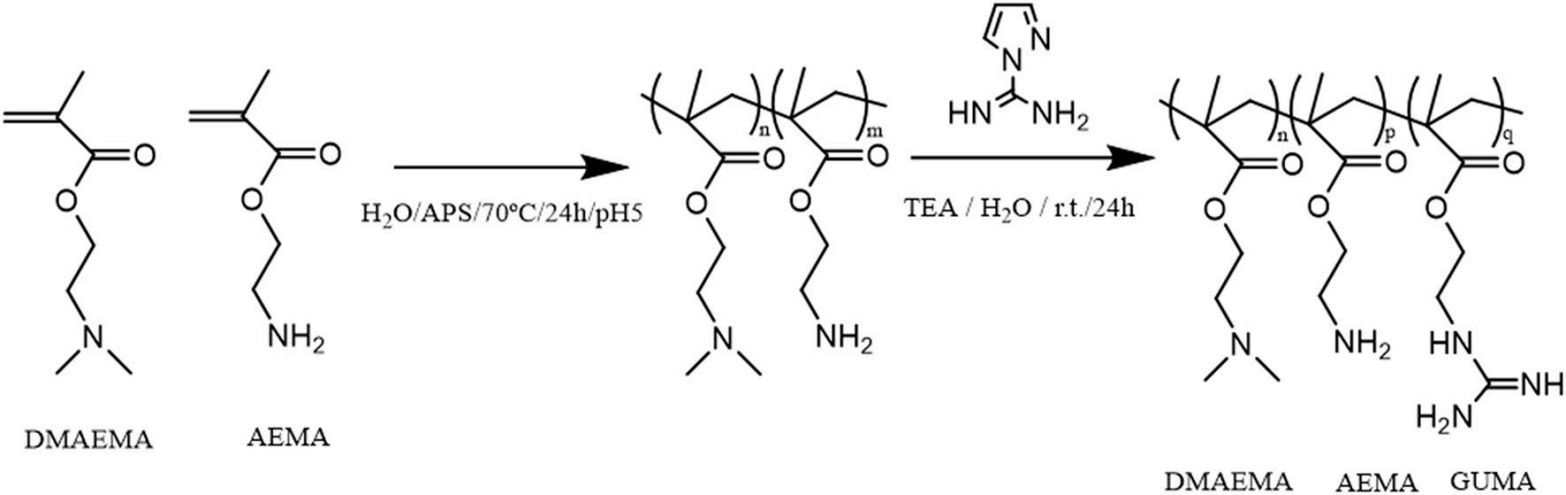
Scheme of the reaction synthesis of the (co)polymers based on DMAEMA and/or AEMA and subsequent modification of part of the primary amino groups into guanidine moieties (GUMA).

(Co)polymers of 2-(dimethylamino)ethyl methacrylate (DMAEMA) and/or 2-(aminoethyl) methacrylate (AEMA) were synthesized by free radical polymerization to obtain polymers containing either primary (AEMA) and/or tertiary amine groups (DMAEMA). The obtained polymers were further functionalized by reacting part of the primary amines with 1H-pyrazole-1-carboxamidine hydrochloride (HPC) to yield copolymers with guanidine groups as shown in the scheme of Figure 1 using earlier described guanylation protocols (Mattheis et al., 2013; Guo et al., 2015). An overview of the synthesized polymers is given in Table 1. The polymer synthesis and guanylation reaction conditions can be found in Supplementary Table 2.

The resulting polymers were characterized by ^1^H-NMR spectroscopy (Table 2). The peaks assigned to the methylene groups neighboring the amino groups in DMAEMA (δ = 3.50 ppm) and AEMA (δ = 3.33 ppm) were used to determine the molar fractions of each cationic group in the polymers. Post-functionalization by guanylation led to a shift of the previously identified peaks (DMAEMA (δ = 3.13 ppm) and AEMA (δ = 2.96 ppm) and the emerging peaks of the methylene groups linked to the guanidine groups (δ = 3.50 ppm) were identified. The guanylated moieties in the polymers will be denoted as GUMA (Guanylated-methacrylate). The experimental molar fractions of the different moieties, DMAEMA, AEMA, and GUMA were calculated by comparison of the integral intensities of the assigned protons (Table 2). The analysis of the obtained polymers by FTIR spectroscopy confirmed the chemical structure (Supplementary Figure 1). The bands at 1,722 and 1,469 cm^−1^ are attributed to the vibrations of the primary and tertiary amino functionalities in AEMA and DMAEMA and the relative intensities for these bands correlate to the chemical composition of the (co)polymers. After modification of the (co-)polymers, a new band from GUMA appeared at 1,676 cm^−1^ corresponding to the stretching vibration of C = N, demonstrating the introduction of the guanidine group. The molecular weight of the synthesized polymers was determined by SEC analysis and is shown in Table 2.

**Table 2:** Polymer characterization.

**Polymer**	**Experimental molar fraction**		**Mn^b^ (g·mol^−1^)**	**PDI^b^**	**Guanylated polymer**	**Experimental molar fraction**		
	f_DMAEMA_^a^	f_AEMA_^a^				f_DMAEMA_^a^	f_AEMA_^a^	f_GUMA_^a^
PAH	–	1	82	2.86	PAG 87H	–	0.13	0.87
					PAG33	–	0.67	0.33
PAL	–	1	45	1.41	PAG 87L	–	0.13	0.87
PD	1	–	96	1.77				
PDA48	0.52	0.48	109	1.95	PDAG18	0.52	0.30	0.18
PDA70	0.30	0.70	127	1.64	PDAG38	0.30	0.32	0.38

*Polymer characterization: experimental molar fraction (f_n_), molecular weight (Mn), polydispersity index (PDI) and mass per charge (M/C) (g·mol^-1^)*.

a *Obtained from ^1^H-NMR analysis*.

b *Obtained from SEC analysis*.

### Polymer-dsRNA complexation and *ex vivo* degradation assays

Polymeric gene delivery complexes are based on the electrostatic interaction between cationic polymers and the negative charge of nucleotides. Upon complexation (polymer-dsRNA complex), the dsRNA gets neutralized and condensed into particles that protect it from the biological environment. The condensation of the dsRNA was investigated by agarose gel electrophoresis. Most tested polymers were shown to form complexes with the dsRNA at N/P ratio 2:1, except for PAG33, PDAG18, and PDAG38, which only fully complexed at N/P ratio 4:1 (Supplementary Figure 2).

As demonstrated in the gel electrophoresis assays, all polymers can form complexes with the dsRNA. The particle size and zeta potential of the formed polyplexes was studied by DLS and zeta measurements. Zeta-potential is used in this work as an indication of the complexation process between the polymer (positively charged) and the nucleic acid (negatively charged). A positive zeta-potential indicates full neutralization of all dsRNA. Focusing on the polyplexes containing guanidine (PAG87H, PDAG33 and PDAG38), the results showed a particle size below 350 nm in all cases (Supplementary Figure 2). The zeta potential analysis results were consistent with the gel electrophoresis assays as full complexation happened at N/P ratios higher than 1.5 (Supplementary Figure 3).

To investigate whether the synthesized polyplexes provided protection against nucleolytic degradation of dsRNA, an *ex vivo* assay was developed whereby gut juice from the lepidopteran *S. exigua* was collected and the dsRNA polyplexes were incubated in this gut juice for different time periods. These *ex vivo* experiments were performed using a 4:1 N/P ratio (Supplementary Figure 4) and at different pH conditions, to investigate the role of pH in the protection ability of the polymer. After incubation, the dsRNA polyplexes were decomplexed and the naked dsRNA was run on a 1.5% agarose gel to observe the stability. The gel pictures are shown in Supplementary Figure 4. At pH 7.5, all polymers were able to protect the dsRNA from degradation in the *S. exigua* gut juice for over 2 h, while naked dsRNA was completely degraded. In fact, preliminary experiments showed that naked dsRNA is degraded after 10 min already (data not shown). At pH 11, only PAG87, the polymer with the highest guanidine content, was still able to protect the dsRNA (Supplementary Figure 4).

**Figure 2:**
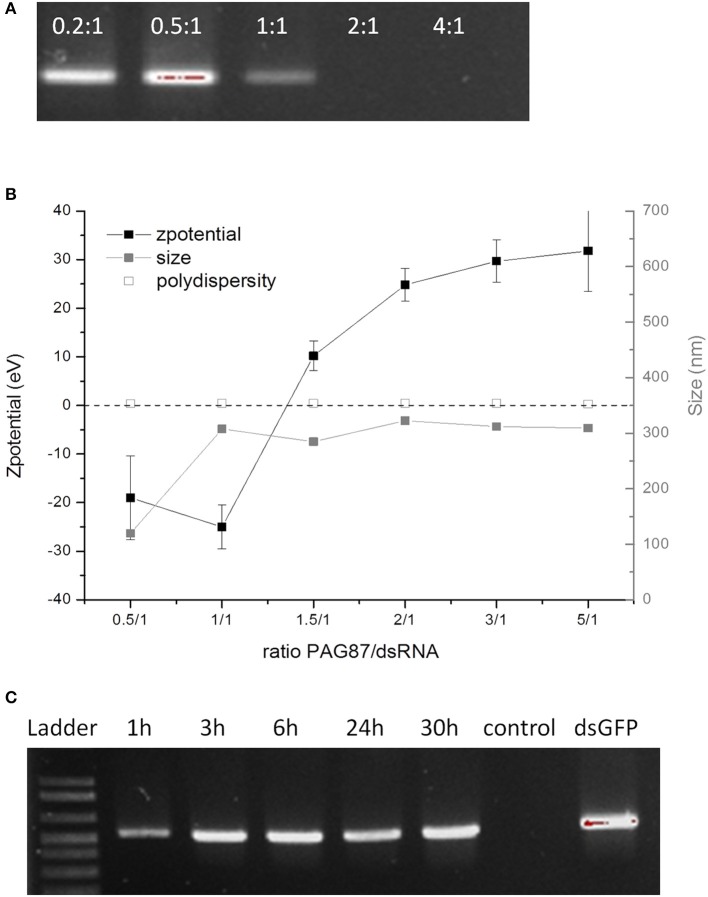
Characterization of activity of PAG87L and ability to protect dsRNA against nucleolytic degradation. **(A)** Complexation of the polymer with the dsRNA at different polymer:dsRNA charge ratios (0.2:1, 0.5:1, 1:1, 2:1, and 4:1) **(B)** Zeta-potential and size of the polyplex for different polymer:dsRNA charge ratios. **(C)** DsRNA polyplexes were incubated in collected gut juice for different time periods (1–30 h) to investigate the stability of the dsRNA when complexed with the polymer. The control represents naked dsRNA incubated in the gut juice for 1 h.

Since at pH 11 only PAG87H could protect the dsRNA, we attempted to further improve it. PAG87H had a high molecular weight, which could possibly be a concern for cellular uptake efficiency. Therefore, we synthesized a lower molecular weight version of this polymer, named PAG87L and further characterized it (Figures 2A-C). Figure 2A shows the complexation of dsRNA and PAG87L at different N/P ratios, showing full complexation between a 1:1 and 2:1 charge ratio. Figure 2B provides confirmation of the complexation, showing a transition from a negative to a positive zeta potential between ratios 1.5:1 and 2:1. Finally, the *ex vivo* degradation assay was repeated for PAG87L, showing stability of the dsRNA in gut juice at pH 11 for at least 30 h (Figure 2C).

### Formulation of dsRNA with PAG87L increases RNAi efficiency in *S. exigua*

**Figure 3:**
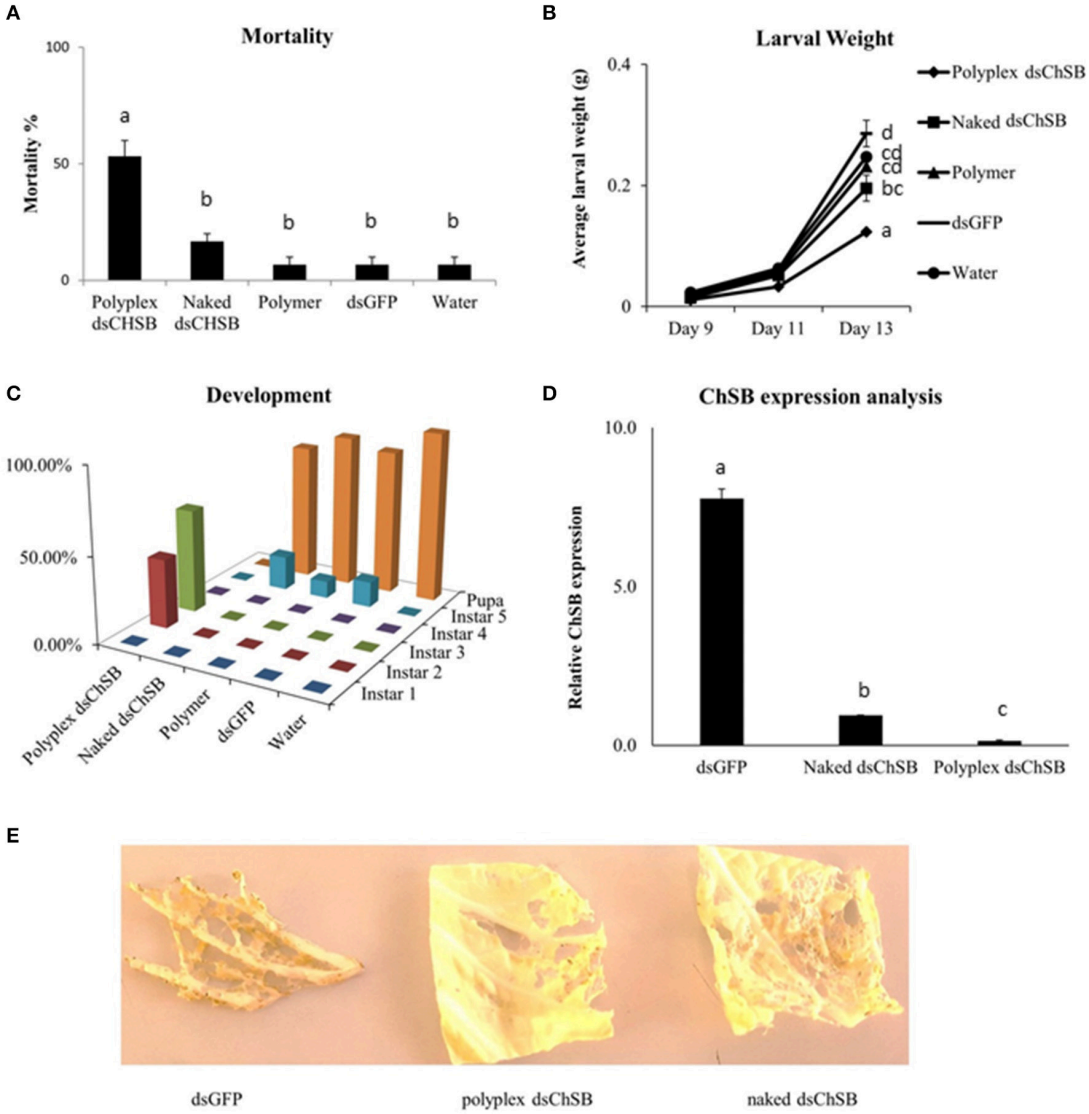
Effect of Chitin Synthase B RNAi silencing on survival, growth, development and transcript silencing of *Spodoptera exigua* larvae. L1 larvae were placed on Chinese cabbage leaf disks coated with dsRNA specific for chitin synthase B. 100 μL of a 500 ng/μL concentration of naked dsRNA was compared with the same amount of dsRNA complexed with the PAG87L polymer at a 4:1 N/P ratio. Ten larvae were placed on each leaf. Water, polymer and dsGFP treatments were used as controls. Error bars represent SEM. **(A)** Mortality of the different treatments after 13 days of feeding on treated leaf disks. A significantly higher mortality was observed for the dsChSB:PAG87L polyplex treatment compared to the other treatments (*P* < 0.05). The experiment was repeated three times (*N* = 30 per treatment for each repeat). Letters above the bars represent statistical significance. **(B)** Average individual weight of the larvae at different timepoints during the experiment. The results indicate a retarded growth (±50%) for the dsChSB:PAG87L polyplex treatment compared to the control treatments. The experiment was repeated three times (*N* = 30 per treatment for each repeat). Letters on the right of the treatment lines represent statistical significance. **(C)** Developmental rate of the larvae over the different treatments indicating a stunted development for the larvae in dsChSB:PAG87L polyplex treatment group, compared to the other treatments. Data on development was obtained for two repetitions of the experiment (*N* = 30 per treatment for each repeat). **(D)** ChSB expression analysis after RNAi silencing. Larvae were fed on dsRNA and dsRNA:PAG87L coated cabbage leaf discs for 5 days before total RNA extraction. Actin, GAPDH, and PGCP were used as reference genes. Data was analyzed by qBase+ and relative expression is given normalized to the reference genes. Letters above the bars represent statistical significance. **(E)** Effects on the herbivory by the caterpillars in the different treatments on Chinese cabbage leaves at day 7.

Feeding bioassays were set up to confirm the functionality of these polyplexes *in vivo*. *S. exigua* was chosen as test organism based on its documented insensitivity to RNAi in association with the strong nucleolytic dsRNA degradation capacity and high gut pH generally observed in lepidopteran insects. Oral RNAi experiments were set up targeting the essential chitin synthase B gene (*ChSB*) wherein mortality, weight and development of the insects were monitored (Figure 3). Feeding on cabbage leaves coated with 100 μL of a 500 ng/μL dsChSB:PAG87L complex resulted in 53.3 ± 6.7 % mortality after 13 days, while naked dsChSB only caused 16.7 % ± 3.3 % mortality. The negative controls, dsGFP, polymer, and water treatments all resulted in 6.7 ± 3.3 % mortality. Statistical analysis, a Kruskal–Wallis test followed by a Games-Howell multiple comparison *post-hoc* test, showed a significant difference between the dsChSB polyplex and the four other treatments (*P* < 0.005), but no significant difference between the naked dsChSB and the control treatments (*P* > 0.05) (Figure 3A). The statistical analysis results are presented in Supplementary Table 3. We recorded an average weight of 124 ± 2.9 mg for larvae in the dsChSB:PAG87L polyplex treatment at day 13, while in the naked dsChSB group, the average larval weight was 196 ± 21 mg. Average weight in the water, dsGFP and polymer treatments were 247 ± 22, 286 ± 6, and 231 ± 7 mg, respectively (Figure 3B). Differences between polyplex dsChSB:PAG87L and naked dsChSB and between polyplex dsChSB:PAG87L and the control treatments were significant (*P* < 0.05), based on a One-way ANOVA test followed by a *post-hoc* Tuckey HSD multiple comparisons test (Supplementary Table 3). The naked dsChSB treatment only resulted in a significantly different weight (*p* < 0.05) with one of the controls, namely the dsGFP control.

The larvae that were feeding on the dsChSB:PAG87L treated cabbage leaves showed a clear developmental retardation compared to those feeding on naked dsChSB or any of the control treatments. On day 4 of the experiment, all larvae had molted into instar 2 in all treatments, except in the dsChSB:PAG87L treatment group, where 30% of these larvae were still in instar 1. At day 10, we could see 50% in L2 and 50% in L3 in the dsChSB:PAG87L treatment. In the other treatments at day 10, all larvae had developed into the L4 stage already. Finally, at day 15, the larvae in the dsChSB:PAG87L were still in the L2-L3 stages (30 and 70%, respectively) while most individuals in the other treatments had already gone into pupation (Figure 3C). To confirm that this increase in mortality was associated with an enhanced RNAi efficiency, quantitative RT-PCR was performed. ChSB expression in the dsGFP and the naked dsChSB treatments was 55- and 6.6-fold higher than in the dsChSB:PAG87L polyplex treatment, respectively (Figure 3D). Differences between the three different treatments were significantly different (*P* < 0.05), based on a One-way ANOVA and *post-hoc* Tuckey HSD test for multiple comparisons (Supplementary Table 3). Representative images of the damage inflicted by the caterpillars in the different treatments to the Chinese cabbage leaves at day 7 are shown in Figure 3E.

### PAG87L formulation increases dsRNA uptake efficiency in CF203 insect midgut cells compared to naked dsRNA

**Figure 4:**
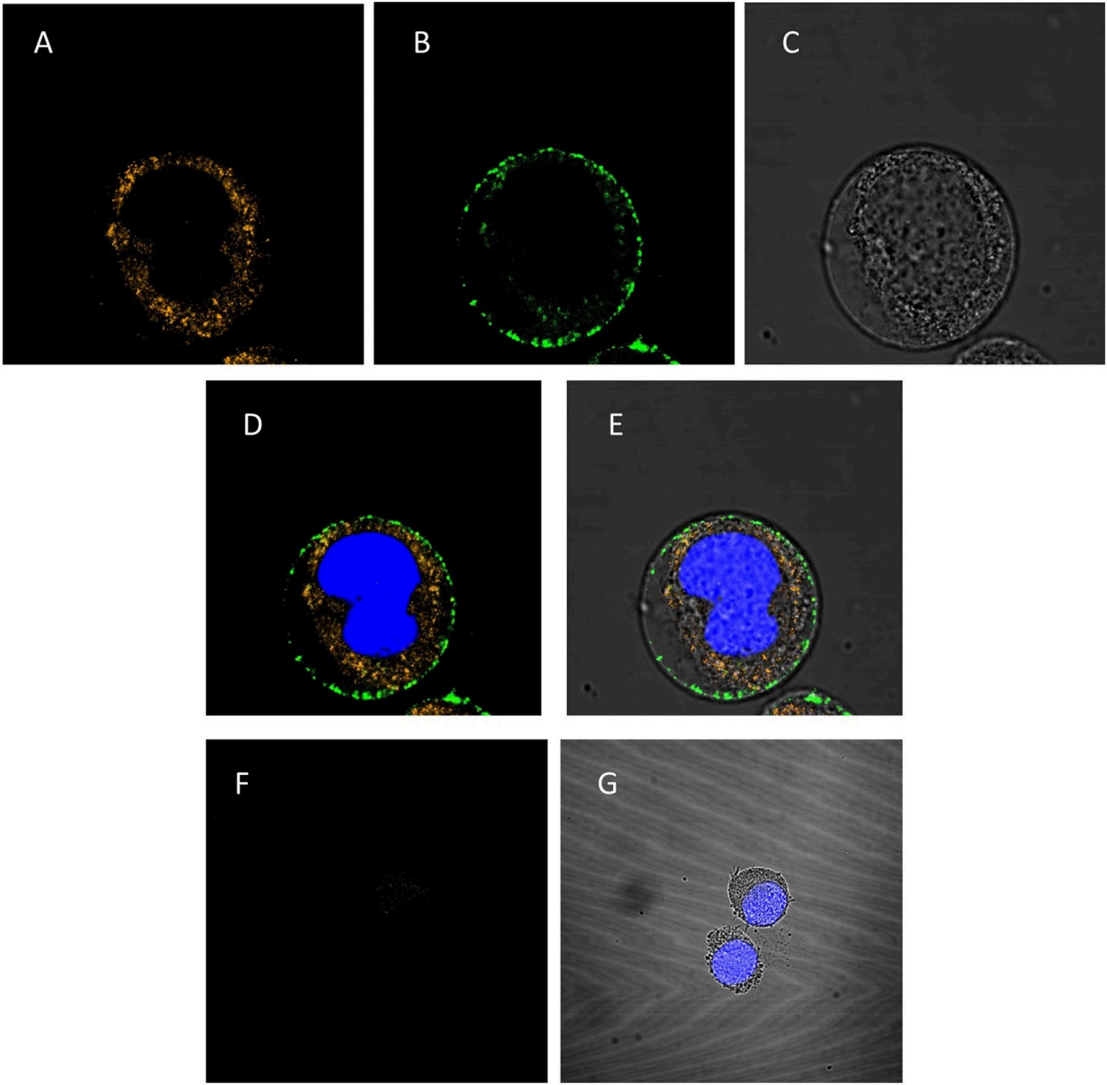
Confocal analysis of cellular uptake of polyplexed dsRNA and naked dsRNA. The nucleus is stained blue with Hoechst® 33342 **(A–E)**: CF203 cells were incubated for 10 min with polyplexes consisting of Cy3-labeled dsRNA and FITC-labeled PAG87L. **(A)** Represents the Cy3-labeled dsRNA visualized using the 549 nm channel, **(B)** represents the FITC-labeled polymer visualized at the 495 nm channel, **(C)** is the image taken under transmitted light and **(D,E)** are merged images of **(A,B)** and **(A–C)**, respectively. **(F–G)**: CF203 cells were incubated for 10 min with naked Cy3-labeled dsRNA. **(F)** Represents the Cy3-labeled dsRNA using the 549 nm channel, **(G)** represents the merged images of the Cy3-labeled dsRNA staining, the Hoechst staining and the transmitted light image.

To investigate whether the PAG87L:dsRNA complexes can be taken up into lepidopteran insect midgut cells, a confocal microscopy study was performed. Figures 4A–E presents the confocal images obtained when polyplexes consisting of Cy3-labeled dsRNA and FITC-labeled polymer were incubated for 10 min in a continuous cell line of lepidopteran *C. fumiferana* midgut cells (CF203). Complete complexation of both labeled molecules was confirmed using gel electrophoresis. The first image demonstrates the uptake of dsRNA in the cells (Figure 4A), while the second channel shows that also the polymer is being internalized in the cell (Figure 4B), mainly concentrated around the borders of the cytoplasm, close to the cell membrane. The third figure displays the image under transmitted light (Figure 4C). Figures 4D,E represent merged channels of both labels and the transmitted light, respectively. Additionally, we repeated this experiment using only naked Cy3-labeled dsRNA (Figures 4F,G). Figure 4F demonstrates that only a very small fraction of the dsRNA is being internalized into the cells, compared to when polyplexes were used (Figure 4A).

## Discussion

Due to major issues such as environmental safety and resistance development to current pest control methods, scientists are constantly looking for novel and intelligent control strategies that limit the potential harm to non-target organisms. The RNAi mechanism has become a promising strategy in this context (Price and Gatehouse, 2008; Huvenne and Smagghe, 2010). Two of its main advantages are its species-specificity and the short persistence of the active molecules in the environment (Dubelman et al., 2014). In the field, RNAi-based pesticides could be applied in different ways. One of the first products that are coming into the market will be an *in planta* application, using transgenic corn which expresses a dsRNA specifically targeting the Western corn rootworm *D. virgifera* (Baum et al., 2007; Bolognesi et al., 2012; Bachman et al., 2013). However, other application methods, such as root absorption, stem injection and conventional spraying of dsRNA have been proposed as well. One major limitation of RNAi is the fact that many insects, especially Lepidoptera, are only moderately sensitive or even insensitive to oral RNAi (Terenius et al., 2011). Several factors involved in RNAi efficiency in insects have been identified and discussed before (Christiaens and Smagghe, 2014; Joga et al., 2016) and dsRNA degradation in the insect is probably the most critical. Notably in Lepidoptera, which comprise some of the most devastating agricultural pests worldwide, degradation of dsRNA in the gut has been shown to happen rapidly (Arimatsu et al., 2007; Liu et al., 2012; Garbutt et al., 2013). This was also confirmed by our results, where we observed complete and rapid degradation of naked dsRNA in *ex vivo* gut juice assays, accompanied with a lack of RNAi silencing when feeding naked dsRNA to the caterpillars. Therefore, for RNAi to be effective against Lepidoptera, formulations will have to be developed to increase the dsRNA's stability inside the insect, at least long enough for the dsRNA to be taken up into the gut epithelial cells where the RNAi process can start.

In this research, we wanted to investigate whether the use of cationic polymers as nanocarriers for dsRNA could represent a functional strategy to increase oral RNAi efficiency and for this study, we used the important lepidopteran pest insects *S. exigua*. Polymers have been shown to be effective carriers for nucleic acids in mammalian systems (Ji et al., 2011; Lin et al., 2013; Cavallaro et al., 2017; Jones et al., 2017), but have so far rarely been used in insects. To the best of our knowledge, only two polymers have ever been used successfully as dsRNA-carriers for oral delivery in insects, namely chitosan in *Aedes* and *Anopheles* mosquitoes (Zhang et al., 2015; Kumar et al., 2016) and a core-shell fluorescent nanoparticle (FNP) in the fruit fly *D. melanogaster* and the Asian corn borer *Ostrinia furnacalis* (He et al., 2013). The latter research consisted of nanoparticles with a fluorescent core of perylene-3,4,9,10-tetracarboxydiimide chromophore (PDI) in the center and polymer shells terminating with multiple amino groups. They demonstrated that these FNPs could pass through the peritrophic matrix and enter the gut cells in *Drosophila* larvae. Feeding dsRNA targeting *CHT10*, a chitinase-like gene, with these FNPs led to a strongly reduced body weight and high mortality after 5 days feeding, while feeding on naked *CHT10* dsRNA only reduced the body weight slightly compared to the dsGFP control and did not lead to a lethal phenotype (He et al., 2013).

The first proof of concept for polymer:dsRNA nanocarriers enhancing RNAi efficiency in insects was published in 2010 by Zhang et al. (2010). They targeted the *chitin synthase* gene in the African malaria mosquito *Anopheles gambiae* using chitosan-coated dsRNA. Chitosan is a linear D-glucosamine and N-acetyl-D-glucosamine polysaccharide which is naturally occurring in nature, namely in the exoskeleton of arthropods. Due to its positive charge, it can associate with negatively charged dsRNA and form a complex. The study did not report on the efficiency of orally delivered naked dsRNA however, making it difficult to assess the actual value of the chitosan nanoparticles. The authors did mention that injection of naked dsRNA was less efficient than feeding the chitosan-coated dsRNA. Recently, Kumar et al. (2016) compared chitosan-coated dsRNA with naked dsRNA and observed at least a 2-fold increase in silencing efficiency and more pronounced phenotypic effects with the former (Kumar et al., 2016). Also, it is unclear how the chitosan really enhances efficiency in mosquitoes. Two hypotheses have been proposed (Zhang et al., 2010). One is an increased stability of the dsRNA in the insects, another is that the nanoparticle might facilitate a more efficient cellular uptake within the mosquito. Especially in Diptera, the uptake efficiency of dsRNA is an important factor explaining the low sensitivity of oral RNAi generally observed in this order (Whyard et al., 2009; Taning et al., 2016). An explanation could be that dipteran insects are missing sid-1-like genes in their genome and rely solely on endocytosis for the uptake of dsRNA (Saleh et al., 2006; Tomoyasu et al., 2008; Cappelle et al., 2016).

Looking at the chemical structure of the polymers used in the above discussed publications, chitosan and polyAEMA, none of them are likely to be suitable for use in very alkali environments, such as the gut of lepidopteran insects, due to the pK_a_ of their amino groups (6.5 and 7.6, respectively). Therefore, we had to look for alternatives. Here, we decided to synthetize new polymers, including some containing guanidine, to remain functional and bound to dsRNA even at an elevated pH. Using free radical polymerization, several (co)polymers based on AEMA and DMAEMA (pK_a_ 9.8) were synthesized. The aim was to use the primary amino groups of AEMA for further modifications by reacting them with HPC to introduce guanidine functionalities (Guo et al., 2015). At the same time, the influence of DMAEMA, a well-known monomer for DNA delivery, with a higher pK_a_, was also analyzed (Christiaens et al., 2005). Different stoichiometric conditions were targeted on the reactions with the primary amino groups to investigate the guanidine content necessary to retain the interaction with the dsRNA.

After synthesis, the polymers were characterized and their functionality was evaluated. Based on our gel electrophoresis analyses, most polymers were shown to complex the dsRNA fully at an N/P ratio between 2:1 and 4:1. Zeta potential experiments also confirmed these observations. When subjecting the obtained polyplexes to degradation assays, all of them protected the dsRNA at pH 7.5 but only the one formed with the PAG87 polymer, which contains the highest amount of guanidine groups (87%), was found to protect the dsRNA against nucleolytic breakdown at pH 11. These results proved the necessity of large amounts of high pK_a_ moieties in the polymer structure so that the interaction with the dsRNA is preserved at high pH. Given that PAG87H was clearly the best performing polymer at pH 11, we continued our functional analyses with this polymer. However, experiments were performed using a lower molecular weight version (PAG87L, Mn = 45 g/mol), since an improved cellular uptake compared to the higher molecular weight PAG87H (Mn = 82 g/mol) was expected based on literature (De Wolf et al., 2007; Synatschke et al., 2011). Analyses demonstrated that PAG87L had the same dsRNA-binding characteristics and protective potential as PAG87H.

The functionality of the polymers was confirmed in an *in vivo* RNAi bioassay, targeting the *chitin synthase B* (*ChSB*) gene in *S. exigua*. Insects generally possess two chitin synthase genes, namely *ChSA* and *ChSB*. The former is involved in the chitin synthesis necessary for cuticle formation while *ChSB* is mainly expressed in the gut and is involved in the production of chitin needed to form the peritrophic matrix, a mesh-like structure lining the gut which has an important role in digesting the food and protecting the gut epithelial cells. Silencing of *ChSB* in our experiments led to decreased feeding of the caterpillars, weight loss, developmental retardation and eventually also mortality in more than 50% of the individuals. Based on these results, *ChSB* might not be the most ideal candidate gene for a crop protection application. While a 90% silencing efficiency at the transcript level led to 50% mortality and a strong retardation of development of the larvae, there might be target genes that have even stronger or faster insecticidal effects. Further research and target gene selection will have to confirm this. In certain coleopteran species, target genes have been identified that lead to near complete mortality within a week at similar RNAi silencing efficiencies. One example is the silencing of Snf7 in the WCR *D. virgifera* (Bolognesi et al., 2012). Whether similar insecticidal effects will ever be achieved for Lepidoptera will however also depend on other factors, in addition to dsRNA stability. In any case, these results clearly confirm the functionality of the PAG87L polymer in significantly increasing the RNAi efficiency and the phenotypic effects this has on the insects.

Through confocal microscopy, we found that both the polymer and the dsRNA are taken up into the cells. We also found that a much higher amount of dsRNA was taken up in the polyplex treatment than in the naked dsRNA treatment, suggesting that the polymers might also increase cellular uptake efficiency. In the polyplex treatment, the dsRNA was found to be present throughout the cytoplasm, while most of the polymer was located around the cell membrane inside the cell, which could indicate that the molecule enters the cell as a polyplex, and dsRNA is then released into the cytoplasm. Whether all the dsRNA seen in the cytoplasm is then processed by the RNAi machinery and contributes to the silencing response remains to be investigated. Additionally, what exactly happens with the polyplex when entering the cell and the polymer after release of the dsRNA is also unclear and requires further study. In mammalian studies, guanidine containing polymers for gene delivery application have been shown to be successful not only due to their ability to bind nucleotides (Funhoff et al., 2004; Song and Chu, 2012; Choi et al., 2015; Guo et al., 2015) but also for enhancing their cell penetrating properties (Treat et al., 2011). Two main dsRNA cellular uptake mechanisms have been proposed in insects. One of these mechanisms works via clathrin-mediated endocytosis (Saleh et al., 2006; Ulvila et al., 2006; Cappelle et al., 2016) and the other is mediated by a *sid-1-like* gene, which encodes a transmembrane channel protein. In nematodes, *sid-1* has been shown to be essential for systemic RNAi and dsRNA throughout the body, except in the gut (Winston et al., 2002, 2007; Feinberg and Hunter, 2003; McEwan et al., 2012). However, the exact role of *sid-1-like* in insects has not been fully elucidated yet. There is also no clear view on whether these two mechanisms are linked, or whether they are completely independent (Cappelle et al., 2016). Further studies on the uptake mechanisms of these polymer-dsRNA complexes in insect midgut cells will elucidate how exactly the guanidine-containing polymers affect the cellular uptake of the dsRNA and whether they indeed have the same cell-penetrating capacity as in mammals (Treat et al., 2011).

Based on this research, we can conclude that the high guanidine-containing polymer PAG87L is able to significantly increase oral RNAi gene silencing in *S. exigua*, which led to a 3-fold increase in mortality and a developmental halt when the essential gene *ChSB* was targeted. To further increase mortality, other target genes can be explored and further optimizations to the formulation and dsRNA concentration can be investigated. These results demonstrate that using RNAi-based pesticides, with the proper formulations to improve stability and cellular uptake of dsRNA after ingestion, could be a promising tool in the control of lepidopteran pests. Furthermore, these type of delivery vehicles could potentially also be of interest for the delivery of other types of nucleic acids. For example, the system could be used to deliver plasmids containing expression cassettes for Cas enzymes and guide RNA. A recent study in a mammalian cell system has shown that polymer-based carriers could be used to achieve successful CRISPR-Cas gene editing and hereby replace the need for viral delivery systems to Timin et al. (2018). However, the particular polymer described in this study is specifically designed for oral delivery of nucleic acids into the gut epithelial cells. Further studies will have to elucidate whether such polymers could also be used for further systemic transport, for example into the germline.

## Availability of data and material

All data generated or analyzed during this study are available from the corresponding author upon request.

## Author contributions

OC, MT, ZM, MD, PD, and GS: Designed the experiments; OC, MT, ZM, MD: Performed the experiments; OC, MT, ZM, MD, PD, and GS: Analyzed the data; OC and MT: Wrote the manuscript; OC, MT, ZM, MD, PD, and GS: Edited the manuscript. All authors read and approved the final document.

### Conflict of interest statement

The authors declare that the research was conducted in the absence of any commercial or financial relationships that could be construed as a potential conflict of interest. The reviewer JJS and handling Editor declared their shared affiliation.
