# Supplementary material for: Increased RNAi Efficacy in Spodoptera exigua via the Formulation of dsRNA With Guanylated Polymers
Source: Front Physiol. 2018 Apr 4;9:316. doi: 10.3389/fphys.2018.00316 (PMC5894468; doi:10.3389/fphys.2018.00316)
Supplement: Supplementary file 1 [file Image1.pdf]

Supplementary Fig. S1 FTIR spectroscopy

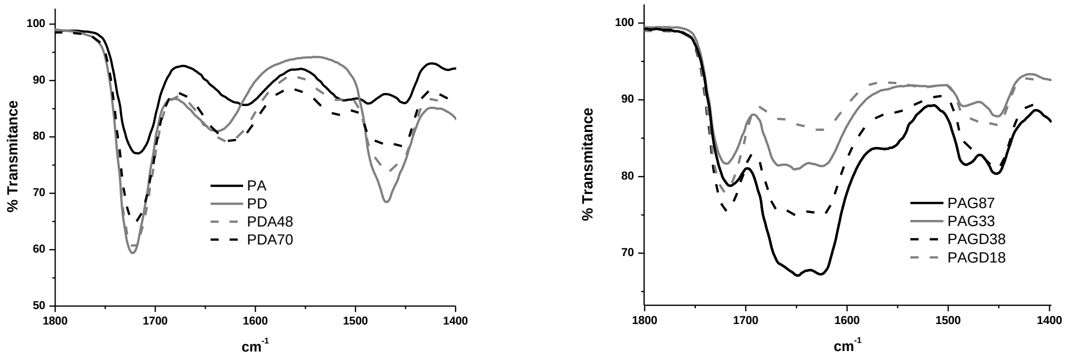

Fig S1. FTIR spectrums of the synthesized (co)polymers Pa, PD, PDA48 and PDA70 (left graph) and the guanylated polymers PAG87, PAG33, PAGD38 and PAGD18 (right graph).
