# Supplementary material for: Increased RNAi Efficacy in Spodoptera exigua via the Formulation of dsRNA With Guanylated Polymers
Source: Front Physiol. 2018 Apr 4;9:316. doi: 10.3389/fphys.2018.00316 (PMC5894468; doi:10.3389/fphys.2018.00316)
Supplement: Supplementary file 2 [file Image2.pdf]

Supplementary Fig. S2: Complexation of dsRNA and synthesized polymers

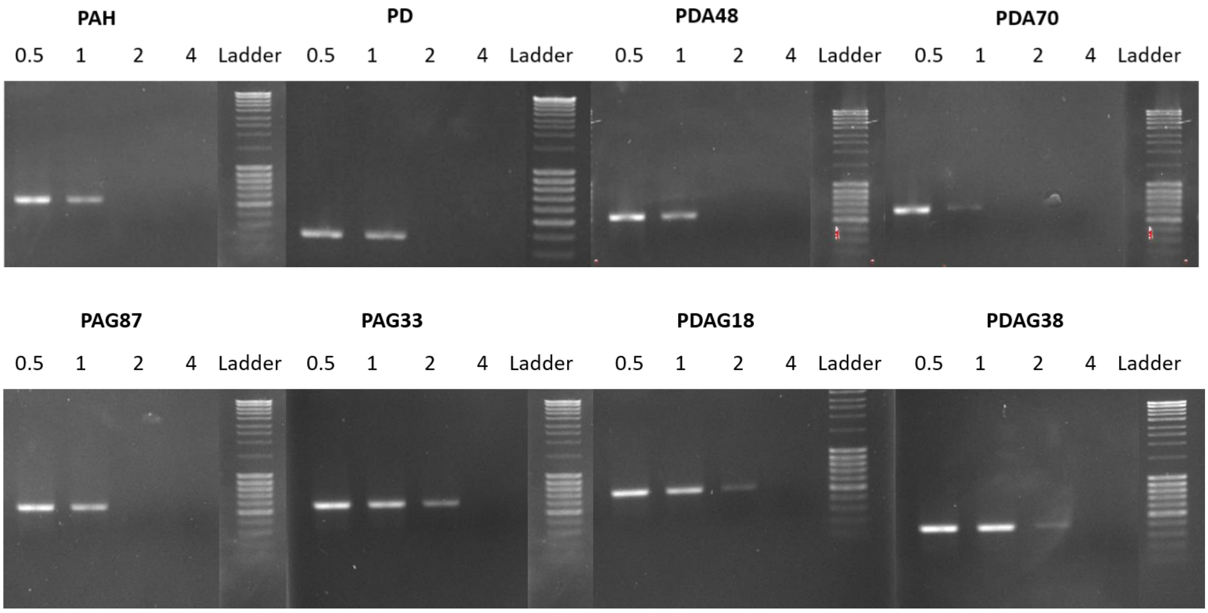

Fig. S2. Overview of complexation of dsRNA with the tested polymers at different N/P ratios (0.5:1, 1:1, 2:1 and 4:1). Visible bands represent dsRNA that is still not associated with the polymer.
