# Supplementary material for: Increased RNAi Efficacy in Spodoptera exigua via the Formulation of dsRNA With Guanylated Polymers
Source: Front Physiol. 2018 Apr 4;9:316. doi: 10.3389/fphys.2018.00316 (PMC5894468; doi:10.3389/fphys.2018.00316)
Supplement: Supplementary file 3 [file Image3.pdf]

Supplementary Fig. S3: DLS measurements of the particle size and Z potential of the polyplexes

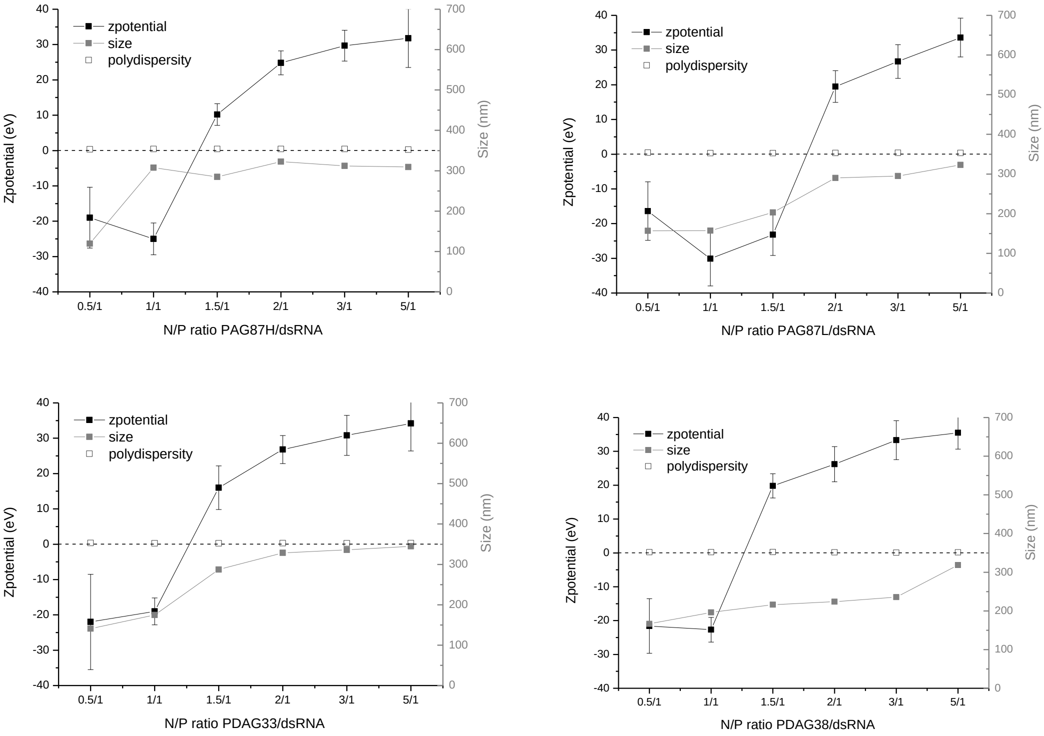

Fig S3. Size and Z potential results for the polyplexes formed with PAG87H, PAG87L, PDAG33 and PDAG38
