# Supplementary material for: Increased RNAi Efficacy in Spodoptera exigua via the Formulation of dsRNA With Guanylated Polymers
Source: Front Physiol. 2018 Apr 4;9:316. doi: 10.3389/fphys.2018.00316 (PMC5894468; doi:10.3389/fphys.2018.00316)
Supplement: Supplementary file 4 [file Image4.pdf]

Supplementary Fig. S4. *Ex vivo* degradation bioassays

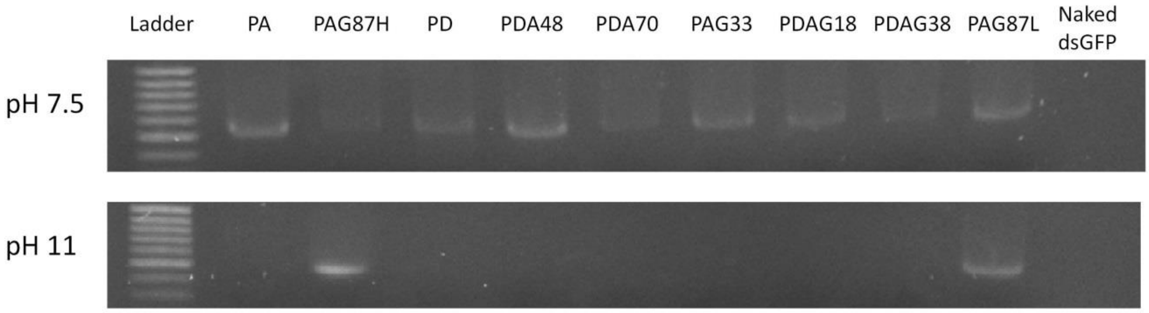

Fig. S4. *Ex vivo* degradation assays. Naked dsRNA or polymer-dsRNA complexes (4:1 N/P ratio) were incubated in *Spodoptera exigua* gut juice for 2 hours. After incubation, SDS was added to stop the degradation reaction and decomplex the polymer-dsRNA complexes. Afterwards, the sample was loaded on a 1.5% agarose gel for electrophoresis. PAG87L is a lower molecular weight version of the PAG87H polymer.
