# Supplementary material for: Increased RNAi Efficacy in Spodoptera exigua via the Formulation of dsRNA With Guanylated Polymers
Source: Front Physiol. 2018 Apr 4;9:316. doi: 10.3389/fphys.2018.00316 (PMC5894468; doi:10.3389/fphys.2018.00316)
Supplement: Supplementary file 5 [file Table1.pdf]

Supplementary Table S1: Overview of all primers used in this study, including primers used for dsRNA synthesis (red=T7 promotor sequence) and primers used for RT-qPCR, for which PCR efficiency and R^2 values are given.

Primer name[1]	Primer sequence	Amplicon size
dsGFP_Fw	TAATACGACTCACTATAGGGTACGGCGTGCAGTGCT	495
dsGFP_Rev	TAATACGACTCACTATAGGGTGATCGCGCTTCTCG	
dsChSB_Fw	TAATACGACTCACTATAGGGAGAGCGTTTCGATGAGGATCAG	403
dsChSB_Rev	TAATACGACTCACTATAGGGAGACTGAGATCGGCAAGTCCATTAG	

Primer name[1]	Primer sequence	Efficiency	R^2	Amplicon size
qPCR ChSB_Fw	GGCATTGCGTTACTGGTCTT	92.00%	0.993	216
qPCR ChSB_Rev	CACTGTTGCCGTTTAGCAGA			
qPCR Actin_Fw	TGCGTGACATCAAGGAGAAG	90.30%	0.997	174
qPCR Actin_Rev	AGAAGGAAGGCTGGAAGAGG			
qPCR GAPDH_Fw	GACAACCACTCATCTATCTTCG	96.10%	0.996	173
qPCR GAPDH_Rev	AACATTTATCTCTACAACGCAATC			
qPCR PGCP_Fw	AGGAGAAGGTGCAATGGATG	94.40%	0.992	180
qPCR PGCP_Rev	TTGTCGAGCTCGTTTTTGTG			

[1]Fw refers to forward primer, Rev refers to reversed primer.
