# Supplementary material for: Increased RNAi Efficacy in Spodoptera exigua via the Formulation of dsRNA With Guanylated Polymers
Source: Front Physiol. 2018 Apr 4;9:316. doi: 10.3389/fphys.2018.00316 (PMC5894468; doi:10.3389/fphys.2018.00316)
Supplement: Supplementary file 6 [file Table2.pdf]

Supplementary Table S2. Polymer synthesis: Feed molar fraction (F_n), monomer ([M]) and initiator ([APS]) molar concentration used for the synthesis of the different (co)polymers. Polymer guanylization: reaction equivalents of AEMA, HPC and TEA used for the synthesis of the corresponding guanylated polymers.

**Polymer**	**Feed molar fraction**		**[M]**	**[APS]**	**Guanylated polymer**	**Reaction equivalents**		
	F_{DMAEMA}	F_{AEMA}				AEMA	HPC	TEA
**PAH**	-	1	1	0.87	**PAG 87H**	1	1.5	1.5
	-	1	1	0.87	**PAG33**	1	0.5	0.5
**PAL**	-	1	0.5	0.87	**PAG 87L**	1	1.5	1.5
**PD**	1	-	1	0.87				
**PDA48**	0.50	0.50	1	0.87	**PDAG18**	1	1	1
**PDA70**	0.25	0.75	1	0.87	**PDAG38**	1	1	1
