# Supplementary material for: Increased RNAi Efficacy in Spodoptera exigua via the Formulation of dsRNA With Guanylated Polymers
Source: Front Physiol. 2018 Apr 4;9:316. doi: 10.3389/fphys.2018.00316 (PMC5894468; doi:10.3389/fphys.2018.00316)
Supplement: Supplementary file 7 [file Table3.pdf]

Supplementary Table S3. Detailed statistical data for the mortality, weight and qPCR data of the *in vivo* RNAi feeding bioassay

Mortality data

(I) Treatment	(J) Treatment	Sig.
Polyplex SeChsB	nAKED SEChsB	.063
	Polymer	.033
	GFP	.033
	Water	.033
nAKED SEChsB	Polyplex SeChsB	.063
	Polymer	.359
	GFP	.359
	Water	.359
Polymer	Polyplex SeChsB	.033
	nAKED SEChsB	.359
	GFP	1.000
	Water	1.000
GFP	Polyplex SeChsB	.033
	nAKED SEChsB	.359
	Polymer	1.000
	Water	1.000
Water	Polyplex SeChsB	.033
	nAKED SEChsB	.359
	Polymer	1.000
	GFP	1.000

Weight data

(I) Treatment	(J) Treatment	Sig.
Polyplex SeCHSB	Naked SeCHSB	.034
	Polymer	.002
	dsGFP	.000
	Water	.001
Naked SeCHSB	Polyplex SeCHSB	.034
	Polymer	.442
	dsGFP	.008
	Water	.155
Polymer	Polyplex SeCHSB	.002
	Naked SeCHSB	.442
	dsGFP	.127
	Water	.929
dsGFP	Polyplex SeCHSB	.000
	Naked SeCHSB	.008
	Polymer	.127
	Water	.377
Water	Polyplex SeCHSB	.001
	Naked SeCHSB	.155
	Polymer	.929
	dsGFP	.377

qPCR data

(I) Treatment	(J) Treatment	Sig.
Control	Naked	.000

	Polyplex G23+ChsB	.000
Naked	Control	.000
	Polyplex G23+ChsB	.039
Polyplex G23+ChsB	Control	.000
	Naked	.039
